# Community intervention programmes with people affected by leprosy: Listening to the voice of professionals

**DOI:** 10.1371/journal.pntd.0010335

**Published:** 2022-03-28

**Authors:** Gema Martos-Casado, Carmen Vives-Cases, Diana Gil-González

**Affiliations:** 1 Department of Community Nursing, Preventive Medicine, Public Health and History of Science, Faculty of Health Sciences, University of Alicante, Alicante, Spain; 2 CIBER of Epidemiology and Public Health (CIBERESP), Barcelona, Spain; Johns Hopkins University, UNITED STATES

## Abstract

**Background:**

Community participation and implementing interventions based on the community are key strategies to eliminate leprosy. Health professionals have an essential role as they are a necessary source of information because of their knowledge and experience, as well as their comprehensive perspective of contexts included in the programmes. This study has the aim of analysing the perceptions on the development of programmes with people affected by leprosy from the perspective of professionals that work at different organisations in endemic contexts.

**Methodology:**

A qualitative study was carried out with the written response to an open question questionnaire which was sent by email. The script content was related to positive aspects and difficulties in daily work, participation from the community in activities, contribution to gender equality and programme sustainability. 27 health professionals were interviewed, 14 women and 13 men, all of which belonged to 16 organisations in India and Brazil. Once the content of the interviews was analysed, two main topics emerged: barriers perceived by professionals and proposals to improve the sustainability of the programmes.

**Principal finding:**

Professionals identify barriers related to social stigma, inequalities, gender inequalities, difficulty managing the disease, limited services, lack of resources and lack of community participation. Furthermore, some necessary recommendations were taken into account to improve programme development related to: Eliminating stigma, reaching gender equality, developing adequate and effective services, guaranteeing adequate and quality resources and achieving compassion among professionals.

**Conclusions:**

Although introducing community programmes with people affected by leprosy has a long history in countries such as India and Brazil, there are still several barriers that can hinder their development. Based on the specific needs of the contexts, recommendations are suggested that, with the involvement of all parties and with sensitive approaches towards human rights and gender, they could help to guarantee universal health coverage and the sustainability of said programmes.

## Author summary

Community participation is an essential strategy to fight against neglected tropical diseases such as leprosy. The people involved should be a priority when implementing interventions, both the people affected and their communities and the health professionals involved in the programmes. This research provides information about barriers and recommendations related to the daily work of health professionals who work at organisations with people affected by leprosy in India and Brazil. Those barriers are related to social stigma, gender inequalities, services, resources and community participation. Health workers propose recommendations to reduce these barriers and to guarantee the sustainability of the programmes.

## Introduction

Neglected tropical diseases (NTDs) have a devastating human, social and economic impact on the most vulnerable and poorest populations [1] and leprosy is considered a NTD by the World Health Organisation [2]. Over 200,000 new cases are reported each year in 120 countries all over the world. Brazil, India and Indonesia account for over 80% of these new cases. Continuity of transmission, disabilities and stigmatisation with leprosy are the main obstacles when addressing the disease [3]. Furthermore, in the case of women, children and other vulnerable collectives, leprosy causes a higher impact due to the fact that they live in situations of greater vulnerability that favour a higher burden of the disease and greater discrimination [4–6].

With the slogan “Zero Leprosy”, different international organisations [7–9] have considered new strategies aimed at achieving a world free from leprosy: Halting transmission, preventing disabilities and promoting inclusiveness. These lines of action also potentially contribute to achieving Sustainable Development Goals 3 (SDG3), that indicates: “Ensure healthy lives and promote well-being for all at all ages” and, in particular, SDG 3.3 that refers to eliminating neglected tropical diseases [10].

Therefore, the importance of implementation research and also based on the community is evident, both focused on improving efficacy and effectiveness (interventions) regarding prevention, elimination and eradication of NTDs in general [11–13] and leprosy in particular [7–9,14].

Thus, community participation, promoted by Alma Ata [15], continues to be a priority in the most recent strategies focused on fighting against NTDs [12] and against discriminating those affected by these diseases [4], therefore, promoting the idea of involving affected people and their communities in making decisions regarding their health and well-being.

In this field, we have not found evidence in literature that examines barriers from the perspective of health professionals in leprosy contexts in order to contrast out results. Nonetheless, research has been found in reviews of literature[16,17] or that analyse these barriers from other perspectives, such as that of people affected by leprosy [18–20] or the community [21]. Results have also been found in other contexts [22] or concerning other diseases, such as tuberculosis or HIV [23].

The different policies and intervention proposals outline the strategic role that community health professionals and agents have to encourage community participation within programmes and contribute to their effectiveness and impact [24–27]. In addition, there is evidence that their professional performance allows to work towards a universal health- coverage, reaching the goals related to SDG3 and address NTDs, including leprosy [12]. The contexts in which the people affected by the different diseases live in order to adapt the strategies to them is essential to guarantee the effectiveness of the programmes. In this sense, health professionals are a necessary source of information to understand the reality, not only because of their experience or specific knowledge on the diseases, but also to gain a comprehensive perspective of the community where the programmes are introduced [24–26].

Therefore, the aim of this study is to explore the perceptions of health professionals regarding the development of programmes with people affected by leprosy.

## Methods

### Ethics statement

The study obtained approval by the Ethical Committee at the University of Alicante (Exp.UA-2019-10-03). Written consent of the people who voluntarily participated in the research was requested, guaranteeing their confidentiality.

### Design

A qualitative study was conducted based on the written response to an open question questionnaire which was sent to participants by email.

### Participants

Organisations from India and Brazil were contacted through the Fontilles Foundation. A representative from this foundation acted as a key informant. A purposive sampling was conducted with professionals who work with people affected by leprosy at organisations or institutions in endemic countries (India and Brazil).

A total of 16 organisations (NGOs, foundations, health centres and public services) accepted to participate in this research. 10 were in India and 6 in Brazil. All participants work in either rural and/or urban settings within the health sector, yet some of them also work in other sectors, such as education, livelihood, occupational health or child protection. Once the organisations had provided their informed consent, the main researcher sent the necessary documentation to take part in the project via the previously established contact.

Finally, 27 professionals belonging to 16 organisations participated in the study. Of all participants, 14 were women and 13 men, aged between 29 and 76 years. They had different professions or positions within the organisation, and they had been working there between seven months and 56 years. 14 of them belonged to organisations in India and 13 in Brazil. In terms of distribution according to gender, five of the 14 people interviewed in India were women, one of which had a management position in the organisation. Of the 13 people interviewed in Brazil, nine were women, four of which had a management position (Table 1).

**Table 1:** Professional profiles of interviewees.

Country	Gender	Age	Position at organisation	Time at organisation
India	W	35	Consultant	No data
India	W	40	Multi-purpose health worker	10
India	W	50	Assistant surgeon	28
India	W	52	Medical Officer	19
India	W	57	Project Officer	26
India	M	50	Professor	17
India	M	33	Medical Officer	4
India	M	40	Co-founder	8
India	M	45	Care and support activities	19
India	M	53	Senior Specialist	10
India	M	54	Co-ordinator	31
India	M	60	Consultant	10
India	M	76	Project Director	56
India	M	No data	Health Educator	No data
Brazil	W	27	Field supervisor	7 months
Brazil	W	29	Supervising nurse	8 months
Brazil	W	32	Nurse	2
Brazil	W	34	Nurse	8
Brazil	W	48	Dermatologist	2
Brazil	W	50	Director	4
Brazil	W	50	Community health professional	24
Brazil	W	54	Leprosy coordinator	12
Brazil	W	57	Technical consultant	10
Brazil	M	53	Doctor	4
Brazil	M	57	Assistance/control	33
Brazil	M	58	Dermatologist	32
Brazil	M	59	Doctor	35

### Data collection

Data were collected through an open question questionnaire based on the conclusions obtained in two prior studies conducted by the research team on leprosy and community [17,28]. Its content was related to the positive aspects and difficulties of the participants’ daily work in leprosy settings, the way affected people and the community participated in the activities organised by the organisation, the way in which the activities organised by the organisation contribute to the gender equity of the target population, and, finally, the key aspects for the sustainability of the programmes.

A section was added that collected sociodemographic variables (gender, age, position in the organisation and years working at it) and technical aspects of the organisations (year of constitution, target population, employee profiles and scope of action). The questionnaire was sent by email between October and December 2019 and the interviewees responded in writing. This method to collect data was used as it was considered to be the most realistic and feasible way due to working circumstances, availability and online platforms.

The open question technical sheet and informed consent were translated into English and Portuguese before sending them to the organisations in India and Brazil, respectively. We asked them for at least one man and one woman to answer open question questionnaire to achieve gender parity, provided that the organisational structure made it possible.

### Data analysis

The answers to the open questions were received via email, therefore, transcription was not necessary. The texts were analysed in line with the thematic content analysis approach, with the aim of classifying the content of the collected data in the texts through category or theme identification that are related to the research objective. The first author grouped the texts and structured them following a process with several stages.

First of all, after repeatedly reading the texts, the elements that were repeated were identified and different categories were created. Some of them were related to open questions and others emerged spontaneously from the content of the texts.

Second of all, a list was created based on 6 main categories: i) social stigma, ii) gender inequalities, iii) difficulty managing the disease, iv) limited services and lack of resources, v) lack of community participation and vi) recommendations by health professionals. The information was initially coded and classified.

Third of all, after this initial coding, two main themes were identified that gave meaning and classified the previous categories. On the one hand, these were “barriers perceived by professionals” including categories i, ii, iii, iv and v. On the other hand, “recommendations and proposals to improve the sustainability of the programmes” included category vi. These topics gave answers, respectively, to two questions posed in the design of the study: What difficulties do health professionals face in their daily work in projects with people affected by leprosy? And, what measures are necessary to put in place to mitigate or eliminate said barriers?

Finally, the recommendations were reclassified and new categories were identified that related previously identified barriers in different typologies.

The whole process was supervised by the other two authors and difficulties or inconsistencies were jointly debated.

## Results

The results are based on two emerging topics and taking into account the participants’ sex and country of origin: perceived barriers and improvement recommendations.

### Barriers perceived by employees

The barriers perceived by employees were identified through the categories: social stigma, gender inequalities, difficulty managing the disease, limited services and lack of resources, and lack of community participation (S1 Table).

### Social stigma

Social stigma is an aspect identified cross-sectionally in all texts. However, in general, interventions with affected communities and people have improved. Professionals in both India and Brazil, regardless of their gender, still perceive stigma as a difficulty in achieving the objectives of the programmes concerning acceptance of the disease.

“*Because of stigma against the disease*, *and the misconceptions about the infectivity of the disease*, *there used to be discrimination against the patient in the family*, *keeping the patient at a distance within the house with a separate bed and utensils*. *Slowly*, *this discrimination could be got over by repeated counselling of the family members”* (Man, India).

This is related to the lack of awareness and knowledge of the disease and misconceptions and local beliefs, as well as laws that still encourage and increase fear and rejection of the disease.

“*(…) There is still social stigma in society even in laws of the nation and expression of the common”* (Man, India).

“*Regarding the social side*, *our society is culturally affected by prejudice and stigma caused by the history of leprosy”* (Woman, Brazil).

They recognise that stigma has a direct impact on the approach to the disease, its acceptance by the affected people and their families, and adherence to treatment.

“*Still taboo is present*, *so most of them are abandoned by families and relatives and the treatment is irregular”* (Woman, India).

“*Stigma of the disease sometimes makes it difficult for those affected and their contacts to handle it”* (Woman, Brazil).

In some cases, this leads them to hide the disease and, in other cases, to reject treatment or seek traditional alternatives which lead to complications and disabilities that directly affect their physical and mental health and, therefore, their quality of life.

“*Few patients refuse to use the footwear due the fear of the stigma and discrimination”* (Man, India).

“*Among the high caste stigma (…)*, *first they try to have traditional superstitious healing”* (Man, India).

### Gender inequalities

Regarding inequalities and gender inequalities, although none of the interviewees explicitly identify them as a barrier, in India they recognise that the inequalities suffered by women in their country are increased by the disease and, therefore, directly influence the approach to it and consequently the programmes.

“*The disease has the potential to trigger changes in the family structure and women affected by leprosy are at a greater disadvantage*, *not only because of the historical gender inequality*, *but also because it is a stigmatised disease”* (Woman, India).

These inequalities directly influence inclusiveness and participation of women affected by leprosy in programmes and accessing services. Furthermore, they are affected by accepting the disease and searching for treatment, which in the case of not getting it, causes the disease to worsen and in many cases isolation and discrimination from their families and communities.

“*Many women experienced marital problems and/or physical abuse*, *regardless of their disability status*. *Women affected by leprosy faced additional problems; related to fear of not getting married*, *fear of separation and poverty*, *and discrimination on account of leprosy”* (Woman, India).

On the other hand, in Brazil, gender inequalities are recognised as a structural phenomenon in society that is evolving towards equality in rights for both sexes.

“*The truth is that in Brazil*, *this aspect is changing quickly (*…*)*. *It is a generational issue*, *sexism tends to a natural and logical disappearance with a more cultured and educated population*. *Equal access to studies*, *equal pay*… *are a reality in Brazil*. *In working at the organisation*. *Other areas of the country are different*. *In my surroundings there are women and men doctors*, *the area manager is a woman and the top managers at state level are also women*…*”* (Man, Brazil).

In general, those interviewed in both countries recognise that activities are being organised to minimise or eliminate the impact of said inequalities at their organisations.

“*Our activities are trying to bring women to the forefront of development initiatives through participatory analysis and planning and leadership and group building sessions (…)* (Woman, India).

“*With proper implementation of the programme*, *creating awareness in the communities and families about specific treatment and definite cure of leprosy*, *this discrimination against women with leprosy has slowly been given up to a large extent*. *At all stages of programme implementation*, *there is gender equality without any discrimination”* (Man, India).

“*(The organisation) is concerned about providing equal opportunities for both sexes*, *such as socio-economic rehabilitation workshops*. *These workshops have always intended to address topics that bring both genders together*, *promoting equal opportunities for both men and women”* (Woman, Brazil).

Finally, some people that were interviewed in Brazil occasionally identify aspects that make accessing services for men difficult and are also addressed by the programmes.

“*At certain times of the year*, *a large amount of male farmers are not in the communities as they go away for weeks to harvest or collect crops*, *this can also happen with fishing*. *So these men cannot take part in detection campaigns in communities*. *We think that there is an ‘inequality’ of working men in this sense”* (Woman, Brazil).

### Difficulty managing the disease

Another barrier identified by professionals is related to the difficulty in managing the disease. This barrier is associated with stigma, physical and psychological effects of the progression of the disease, rejection of treatment, difficulty in monitoring affected people, their socio-economic status and the context in which they live.

“*A low level of education of patients and relatives makes it difficult to understand the diagnosis*, *treatment and prevention of disabilities*, *it means the patient delays looking for diagnosis from health services*. *Prejudice and stigma increase due to the lack of information and the difficulty to access it”* (Man, Brazil).

If leprosy is not diagnosed early and there is no adequate follow-up of treatment, it causes loss of strength or sensation, deformities, ulcers and leprosy-reactions. Those consequences cause limitations and disabilities that affect both their social and family relationships, as well as work.

“*Treating reaction and nerve damage in leprosy can be very frustrating sometimes*. *It is still very difficult to predict which patients will go in reaction and who will develop nerve damage and deformity”* (Man, India).

“*Loss of sensation in hands and feet imposes severe limitations on the leprosy affected person in their routine job”* (Man, India).

Furthermore, physical effects directly influence psychological effects, as does social stigma. Health professionals identify depression and anxiety as psychological effects, recognising them mainly in women and girls, in general.

“*Due to the vast distance many families have to travel to reach the centre*. *Due to the lack of manpower*, *we are not able to reach remote areas and their communities to bridge in education and communication as the women and children experience more stigma and mental health issues than men”* (Woman, India).

In terms of rejecting treatment, they express reasons such as fear of being stigmatised or discriminated; also not considering the initial symptoms as symptoms of the disease, and therefore, they consider treatment is not necessary.

“*For the general community*, *leprosy then meant presence of gross deformities*. *The presence of patch/patches on skin could never be considered as signs of leprosy by the people*. *Diagnosis of leprosy was not accepted in most cases*. *Treatment too was refused then”* (Man, India).

They link the difficulty of monitoring treatment with socio-economic status and the context in which people affected by leprosy and their families live, which are usually environments of poverty and with low levels of education. This situation forces people to find jobs elsewhere, making access to services and treatment adherence difficult.

“*As Mumbai is a metropolitan city a lot of our patients migrated from the endemic states*. *They come to Mumbai in search of work*, *they get temporary work if the work stops*, *they moved to different area in search of work*. *In this kind of floating population it becomes difficult to hold on to patients for a long time and treatment adherence is poor in this population”* (Man, India).

“*Economic conditions of those affected are a treatment barrier”* (Woman, Brazil).

In many cases, they live in remote and tribal areas that make accessing health services difficult. They must travel long distances and invest a lot of money to get there. Furthermore, transport is a problem due to the poor infrastructure or weather conditions that make the surrounding conditions even more difficult. An example of this is some rural areas in India and Amazon rainforest areas in Brazil.

“*We see very few local people*: *Most are immigrants from other parts of India which are under development*. *These areas are inhabited by tribal people and socio-economically backward communities where there is less penetration of health services (…)*. *It is difficult to convince them they need treatment for 6 to 12 months (…)”* (Man, India).

“*In the rural Amazon areas*, *the main problem is logistics*. *Transport is almost exclusively fluvial*, *there are many dispersed communities*, *long distances and the communication routes depend on weather factors”* (Woman, Brazil).

### Limited services and lack of resources

The limited services and lack of resources, both human and economic, are identified as aspects that hinder programme development. In some cases, the lack of investment from the government to support programmes is identified. In addition, this includes the lack of resources for laboratory diagnosis, approaching reactions and providing self-care guidelines (including the adequate adapted shoes), as well as attention for remote communities.

“*In the communities and small riverside cities of the Amazon*, *not only are health resources scarce*, *but there are also practically no state*, *orthopaedic and rehabilitation resources*. *People already cured of leprosy but with long-term effects do not have access to the correct shoes*, *clothing*, *adapted utensils*. *Many of them do not know the basic practices of added damage prevention (*…*) local health professionals are poorly trained in this sense”* (Woman, Brazil).

“*Diminishing leprosy trained experts in government and private health facilities for diagnosis (and) treatment of complication in leprosy”* (Woman, India).

Lack of trained professionals, rotation and lack of interest from some of them are expressed as aspects that hinder programme continuity.

“*Doctors and paramedic staff not taking an interest in leprosy programme”* (Man, India).

“*In the town we still have a large rotation of medical professionals that makes it difficult to monitor* cases, many are trained and soon move to other locations, leaving the municipality unassisted” (Woman, Brazil).

Added to this is the lack of multidisciplinary teams and the lack of adequate specialised services to refer patients, either due to the lack of commitment from the public sector or the lack of leprosy experts in local centres.

“*(…) Every other patient is referred to our centre for diagnosis*, *reaction management*, *skin smear test*, *wax therapy*, *muscle stimulation which could have been managed locally*. *In absence of these services*, *patients are travelling 400 to 500 kilometres (KM) to our centre”* (Man, India).

*"Vulnerability in the support of the public sector*, *which due to various reasons*, *results in a late diagnosis*, *the signs and symptoms of the disease are not properly disclosed*, *even in hyperendemic areas”* (Woman, Brazil).

### Community participation

In terms of community participation, on the one hand, most interviewees mention in one way or another the programmes as a positive aspect. Among others, they mention their participation in the formation of self-care groups, in the association of people affected by leprosy to fight for their interests, in the mobilisation of community resources, in the leadership of the organisation’s activities and in socio-economic reintegration initiatives.

“*The organisation conducts regular meetings with the patients and their family members*. *On special events we involve the patients in the Health Education programme and deformity care services*. *The community is involved in resource mobilisation for livelihood programmes*. *Education support for the children and grocery support to the patients and their family*. *The community actively participates in the awareness programmes and health camps”* (Woman, India).

“*Morhan- Movement of Reintegration of People Affected by Leprosy- through its local representation*, *has carried out some interventions to qualify the care of people affected by leprosy*, *especially those with physical long-terms effects*. *They act on a political level to guarantee the rights of those with physical disabilities and special needs*, *as well as working towards reducing prejudice and social stigma*” (Man, Brazil).

On the other hand, a minority group of workers interviewed in India and Brazil identify a lack of community participation as a barrier to carry out the programmes. In some cases, they mention the difficulty to involve all social groups and community leaders in programmes, yet without mentioning the reason behind this.

“*Difficulties in involving all social groups (such as religious and political leaders) responsible for promoting respect for human dignities in educating communities”* (Woman, India).

Others mention participation as a mere individual aspect related to accepting services or adherence to treatment, which cannot be considered as community participation:

“*In relation to leprosy cases in the area*, *patients who are treated generally collaborate and participate in their treatment*” (Woman, Brazil).

### Recommendations

Recommendations emerge from the interviews that the workers identify as necessary to guarantee the continuity and sustainability of the programmes. They have been categorised, some of which are related to the barriers that have already been mentioned (Table 2).

**Table 2:** Recommendations by professionals to guarantee continuity and sustainability of programmes.

■ Zero stigma a. Include leprosy as a priority on political agendas b. Strengthen awareness on the disease c. Reform the national legal framework related to leprosy ■ Gender equality d. Create strategies and interventions aimed at including and empowering women with leprosy e. Outline the role of leprosy workers and women caregivers f. Offer the same work and salary opportunities g. Motivate women to participate in politics and decision-making bodies h. Allocate resources and generate evidence for inclusion ■ Managing leprosy and its complications i. Reinforce active search and early diagnosis j. Guarantee accessible treatment and comprehensive facilities k. Prioritise preventing disabilities l. Implement socio-economic rehabilitation interventions ■ Adequate and quality services and capacity building m. Guarantee quality care, services and infrastructures n. Guarantee technical training and professional replacement o. Promote multidisciplinary integration and access to specialised care p. Reinforce strategies to enable resources and financial support q. Strengthen community participation r. Create networks and alliances ■ Principles and values s. Promoting compassion among health professionals

Said recommendations are related to the intervention proposal aimed at i) reducing stigma through community awareness or legal reforms, for example; ii) including women and gender equality in programmes, work and decision-making bodies; iii) promote services offered for them to be adequate and effective to reach the goals of “Zero Leprosy” that strengthen interventions, such as actively searching for cases, physical and socio-economic rehabilitation and community participation; iv) guarantee resources, whether material, human and economic, that are adequate, accessible and high quality and, finally; v) include principles and values that recognise promoting compassion among healthcare professionals (S2 Table).

## Discussion

The results of this article show barriers that health professionals identify that hinder the achievement and sustainability of interventions and programmes with people affected by leprosy in India and Brazil. The main findings state that the main areas for improving leprosy interventions in the community are reducing stigma, gender issues, for both women and men, well-designed service interventions, resource provision and promoting compassion among health professionals.

### Social stigma as a cause and consequence of leprosy

This study shows how stigma influences the way in which people affected by leprosy experience the different processes related to their health. Furthermore, it is perceived as a cross-sectional element that affects the other identified barriers, while also being the cause and effect of the mechanisms that perpetuate the disease in endemic countries. This could include, for example, a lack of knowledge, misconceptions, local beliefs and fear of becoming infected [29,30]. These results are in line with existing literature and, in addition, are observed in a wide range of diseases or illnesses, such as other NTDs, mental health, cancer, obesity or other infectious diseases, such as AIDS [31].

Although stigma is identified as a barrier by the interviewed professionals both in India and Brazil, particular realities are observed in each country. In the case of India, stigma because of leprosy, is also mixed with other “interrelated stigmas” [31], such as the caste system or gender inequalities, which, in turn, are supported by laws that discriminate people affected by leprosy. In this sense, despite advances in the last year, there are still 108 discriminatory laws in India, making up for 44% of the laws in the world [32].

In the case of Brazil, although there is still much to be done, other important steps have been taken on a structural level to fight against discrimination against people affected by leprosy. For instance, laws that discriminated people affected by leprosy were abolished. Furthermore, the Assembly voted in favour of the rights of people affected by leprosy and their families; the term leprosy was replaced by *hanseniase* and the national observatory on human rights and leprosy was created to fight against discriminatory institutional practices [33].

The recommendations by the professionals regarding stigma are focused on reinforcing awareness on all levels and including leprosy on political agendas and in legal reforms. These results agree with the urgent need to involve institutions and governments in eliminating discriminatory policies and practices implemented on a structural level [34,35]. In addition, these recommendations are aligned with the document *Principles and Guidelines for the Elimination of Discrimination against Persons Affected by Leprosy and Their Family Members* of the United Nations, which is a roadmap to fight against stigma [4].

### Gender inequalities

The professionals interviewed in India identify gender inequalities to a greater extent and how these affect people affected by leprosy in a particular way. There is sufficient evidence about how the legal framework of the country and the aforementioned caste system directly influence the situation of inequality of women in India compared to other countries [36].

Regarding the results in Brazil, a positive evolution related to an improvement in gender equality can be observed. The cause could be due to different processes. On the one hand, the aforementioned progress in the fight against discrimination of people affected by leprosy, which also includes women in a particular way. On the other hand, cultural and political aspects that have driven the country towards a broader path of social movements with greater participation from women. Feminist movements since 1975 have influenced institutions involved in women’s rights that, throughout the country’s political history, have influenced in one way or another the implementation of public policies with a gender perspective [37,38]. These processes have permeated all areas of the country and have also been reflected in leprosy contexts in which, in line with the findings of this study, a tendency is observed by organisations towards the implementation of interventions with a gender approach, or at least to recognise the need to do so [5].

In general, data currently continues to place women in more vulnerable positions due to feminisation of poverty or discrimination in the workplace [39]. In contexts of leprosy, evidence continues to show how women who suffer or have suffered from the disease have a worse quality of life and are discriminated against or stigmatised to a greater extent than men. This is due to their worse possibilities to access health services and the difficulties to carry out domestic and care roles [6,40]. In order to understand these processes in depth, the impact of public policies and health strategies implemented by public and private institutions should be assessed, particularly those aimed at women and people affected by leprosy.

### Leprosy management, quality services and accessible resources

In line with existing evidence, the results of this study show the factors that hinder managing leprosy, including physical and psychological consequences, socio-economic status or living in remote areas [16–19,21,41]. In order to mitigate this situation, the professionals interviewed propose interventions aimed at detecting cases early, accessing adequate treatment and comprehensive facilities, preventing disabilities and socio-economic rehabilitation [17]. All this coincides with what literature states, except that the professionals interviewed, contrary to what was expected, do not mention recommendations related to mental health [42]

The lack of health professionals and their lack of training are barriers that hinder guaranteeing quality services in leprosy [16–18].

On the one hand, the incorporation of community-health workers into health systems is considered an effective measure in the absence of a specialised workforce [25]. An example could be the Accredited Social Health Activists (ASHA) in India, which, in addition to being a link between health systems and communities, they are involved and play a crucial role in active case detection and leprosy monitoring systems. [43,44]. On the other hand, there is evidence showing that professionals with insufficient training have a negative impact on the diagnosis and management of the disease [26]. Specialised training is required with innovative approaches that facilitate its reach, such as the design of e-learning programmes to train and supervise primary healthcare teams [45] or the use of mobile technologies to help frontline workers facilitate diagnoses.

One of the challenges in leprosy programmes is caring for remote populations, as the results suggest. There are experiences of effective interventions related to the active detection of cases based on the selection and training of local personnel in areas that are difficult to access [46]. Likewise, there are also those based on innovative technologies to identify “hot spots” (areas with a high density of cases) [47,48] or to predict new cases from household contacts [49]. Therefore, in order to ensure universal healthcare coverage for people affected by leprosy living in remote areas, effective evidence-based interventions involving community participation would be necessary.

The results display a lack of economic and infrastructure resources which are barriers that make sustainability difficult. The professionals highlight in their recommendations that there is a need to find new formulas for financing. They agree with the evidence that, in order for them to be sustainable, governments need to be involved, starting with national budgets, with contributions from collaborators [50]. Exploring innovative strategies to access resources would be relevant through, for example, the use of technology, which can be effective in programmes with people affected by leprosy.

### Community participation

The results show a positive value that is given to the participation of those affected and the communities in the programmes. This coincides with the literature that mentions community participation as a key aspect both for the achievement and sustainability of the programmes [15,50]. Despite this, some professionals identify the lack of involvement of certain community leaders, both religious and political. It would be necessary to research which aspects are causing this to occur and clearly recognise the importance of religious and political leaders involvement in accepting and fighting against the disease and eliminating stigma, [21] as evidenced in other stigmatised diseases [41,51].

Likewise, the results show the important role of social movements or associations, such as MORHAN in Brazil, in fighting for the rights of people affected by leprosy. They coincide with the existing evidence about the role of ASHA groups in India that also act from community activism to defend the rights of the affected people [43,44] For future research, it would be interesting to analyse the role of these movements as agents of change, as well as their contribution to improving health, supporting community participation and the sustainability of programmes for people affected by leprosy, their families and communities.

The results also indicate how misconceptions exist in the concept of community participation. In contexts of NTDs, where the role of the community is important to achieve more effective interventions, it is necessary to include the definition of community and clarify specific needs when designing said interventions [52]. Furthermore, establishing which is the expected or desired level of participation in the community would offer different positions regarding involvement in the programme [27].

### Promoting positive attitudes of health workers towards leprosy

Finally, although promoting compassion among professionals is a less referenced recommendation, analysing it is important as it is not mentioned much in international strategies. Interviewees relate it to respect, empathy or motivation towards work. This aspect could be related to fighting against stigma, as evidenced in other publications that show how dehumanising or stigmatising attitudes or certain behaviours by health professionals can negatively influence adherence to treatment or have a large impact on people affected by leprosy [19,21]. Thus, as demonstrated in this study, strategies are needed that are aimed at promoting those principles and values to offer more compassion among professionals a more humanised care and that breaks the barriers of behaviours that, from the organisations, help to perpetuate the stigma of people affected by leprosy [35].

### Limitations

On the one hand, the limitations are related to data collection via email that could have influenced content saturation of the responses and the non-possibility of clarification or delving into issues during the interviews. On the other hand, comparing results with other research is limited as we have not found any study that specifically analyses the perspective of health professionals who work with people affected by leprosy in endemic countries.

While this article is being written, we are in the middle of the COVID-19 pandemic that is affecting the whole world and more aggressively countries such as India and Brazil. The interviews were conducted a few months before the pandemic, so it would be interesting the reassess the situation of the organisations and employee perceptions after the pandemic and analyse the differences between the two periods of time.

### Strengths

Regarding the strengths of this research, this study provides relevant information for community health intervention clinical practice and public policies by showing the reality experienced by health professionals in leprosy organisations in India and Brazil. It would be appropriate for the people in charge of these organisations and other public institutions or bodies to take these results into account in order to develop or reformulate policies, programmes or interventions aimed at people affected by leprosy.

## Conclusions

Introducing community programmes aimed at people affected by leprosy has seen some progress in India and Brazil. However, according to the professionals involved in these programmes, there are still barriers that can hinder their correct development and their usefulness in improving the lives of people with leprosy. The recommendations by these people with experience, attending to the specific needs of the affected countries, require the involvement of institutions and society, as well as working with approaches that are sensitive to human rights and with a gender perspective. Therefore, these strategies could contribute to guaranteeing healthcare coverage for everyone equally, as well as the effectiveness and sustainability of programmes with people affected by leprosy and their communities.

## Supporting information

S1 Table Barriers identified by health worker´s related with leprosy-programmes development in India and Brazil, 2019. (ZIP)

S2 Table Health workers´ recommendations to improve leprosy interventions in the community in India and Brazil, 2019. (ZIP)
