# Supplementary figures and images for: Community intervention programmes with people affected by leprosy: Listening to the voice of professionals
Source: PLoS Negl Trop Dis. 2022 Mar 28;16(3):e0010335. doi: 10.1371/journal.pntd.0010335 (PMC8989298; doi:10.1371/journal.pntd.0010335)

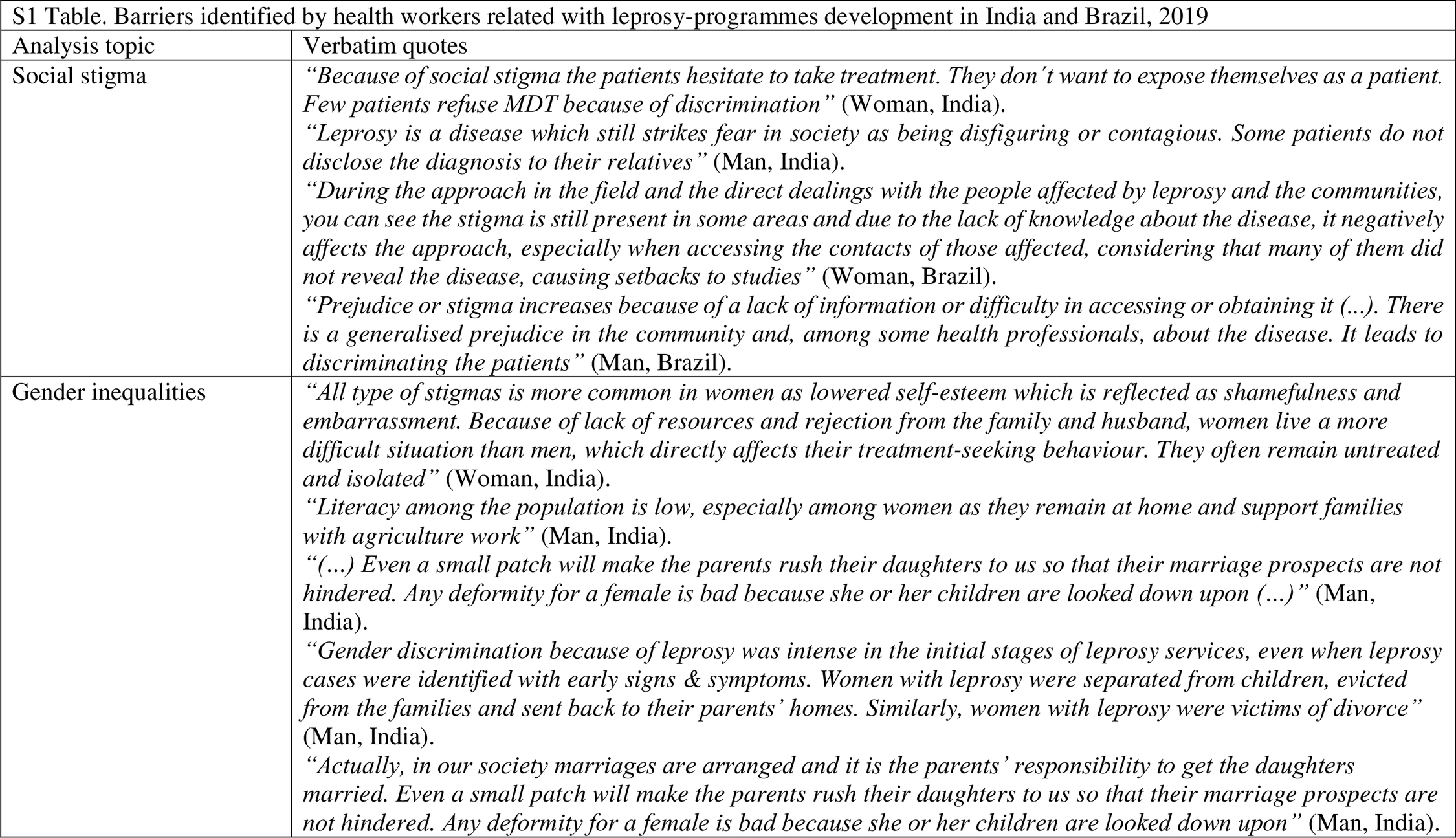

Supplement: S1 Table — (ZIP) [file pntd.0010335.s001.zip › PACE Corrected/S1_Table.tif]

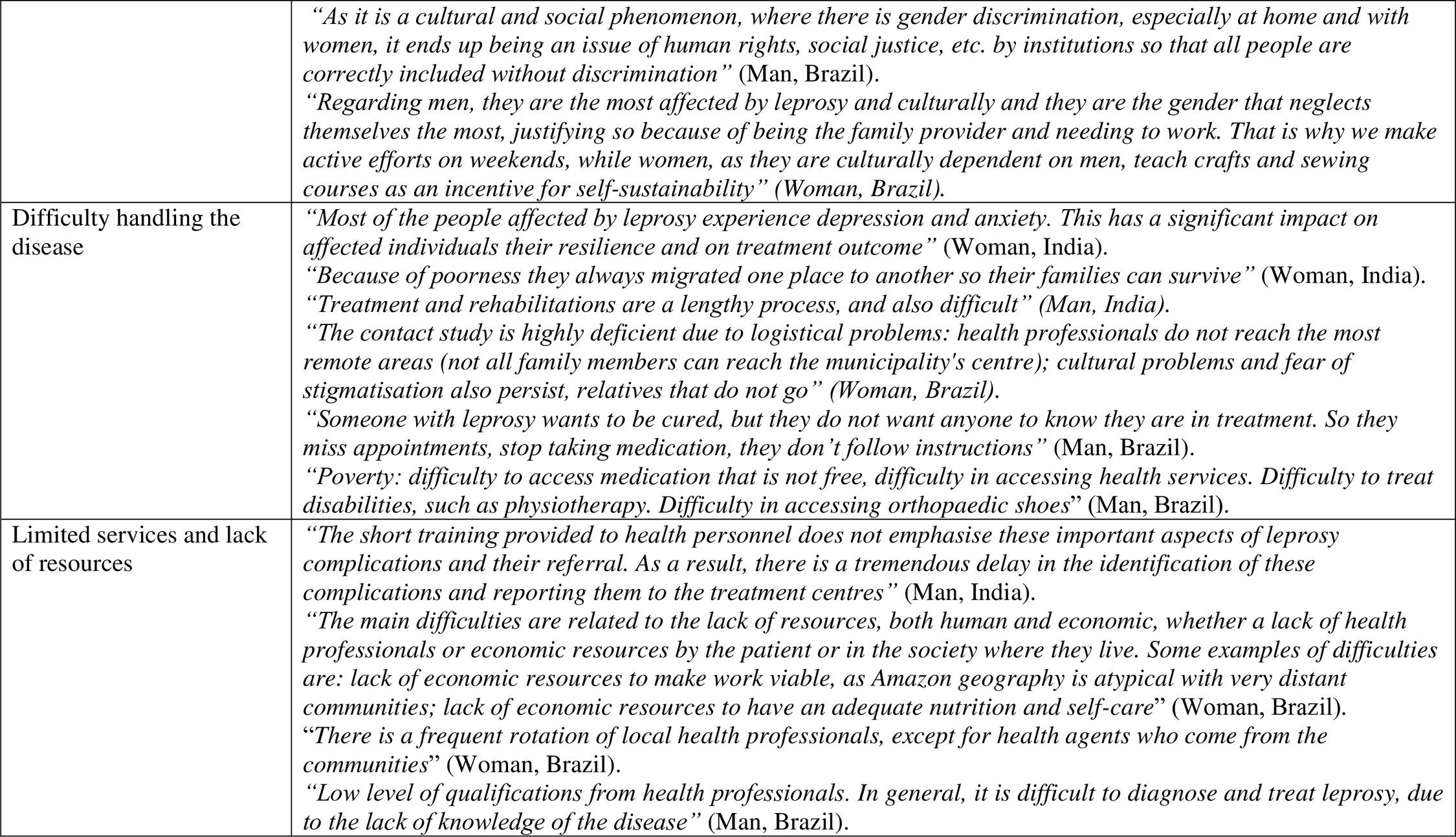

Supplement: S1 Table — (ZIP) [file pntd.0010335.s001.zip › PACE Corrected/S1_Table.tif]

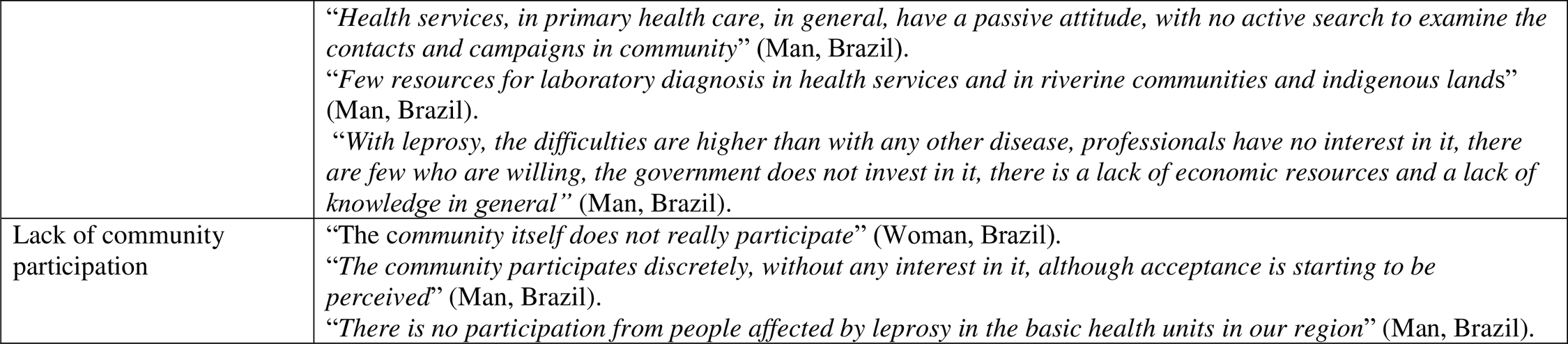

Supplement: S1 Table — (ZIP) [file pntd.0010335.s001.zip › PACE Corrected/S1_Table.tif]

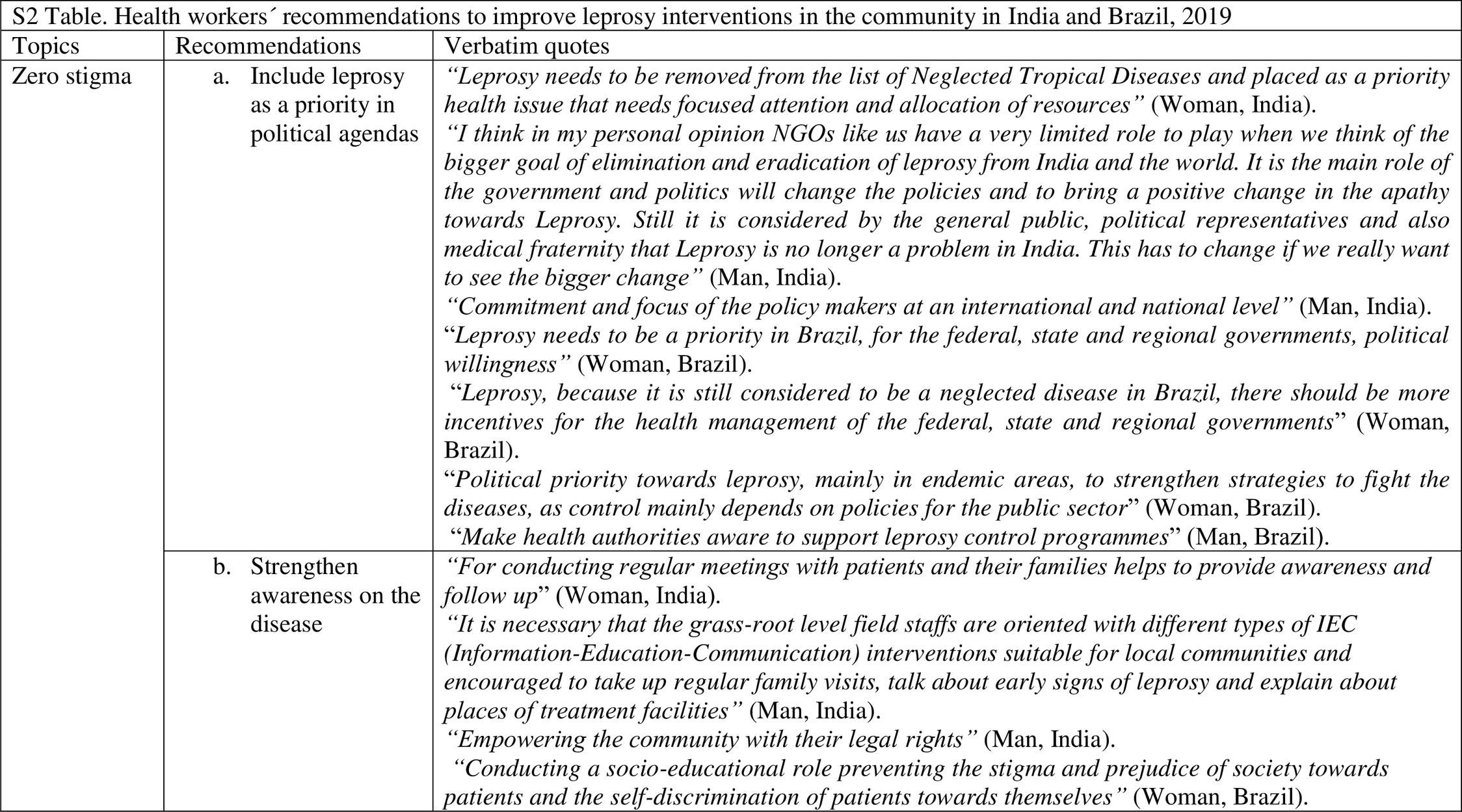

Supplement: S2 Table — (ZIP) [file pntd.0010335.s002.zip › PACE Corrected/S2_Table.tif]

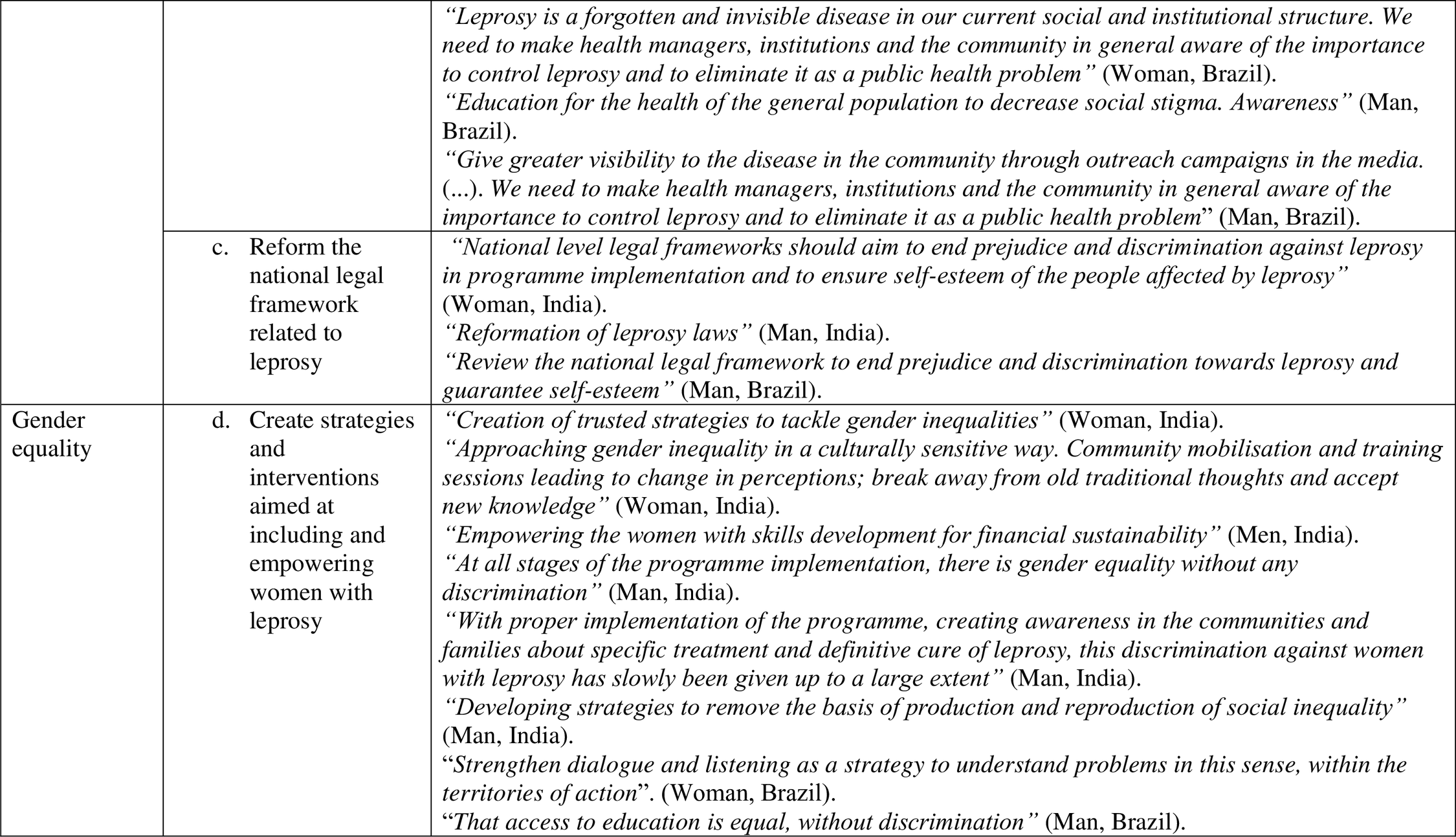

Supplement: S2 Table — (ZIP) [file pntd.0010335.s002.zip › PACE Corrected/S2_Table.tif]

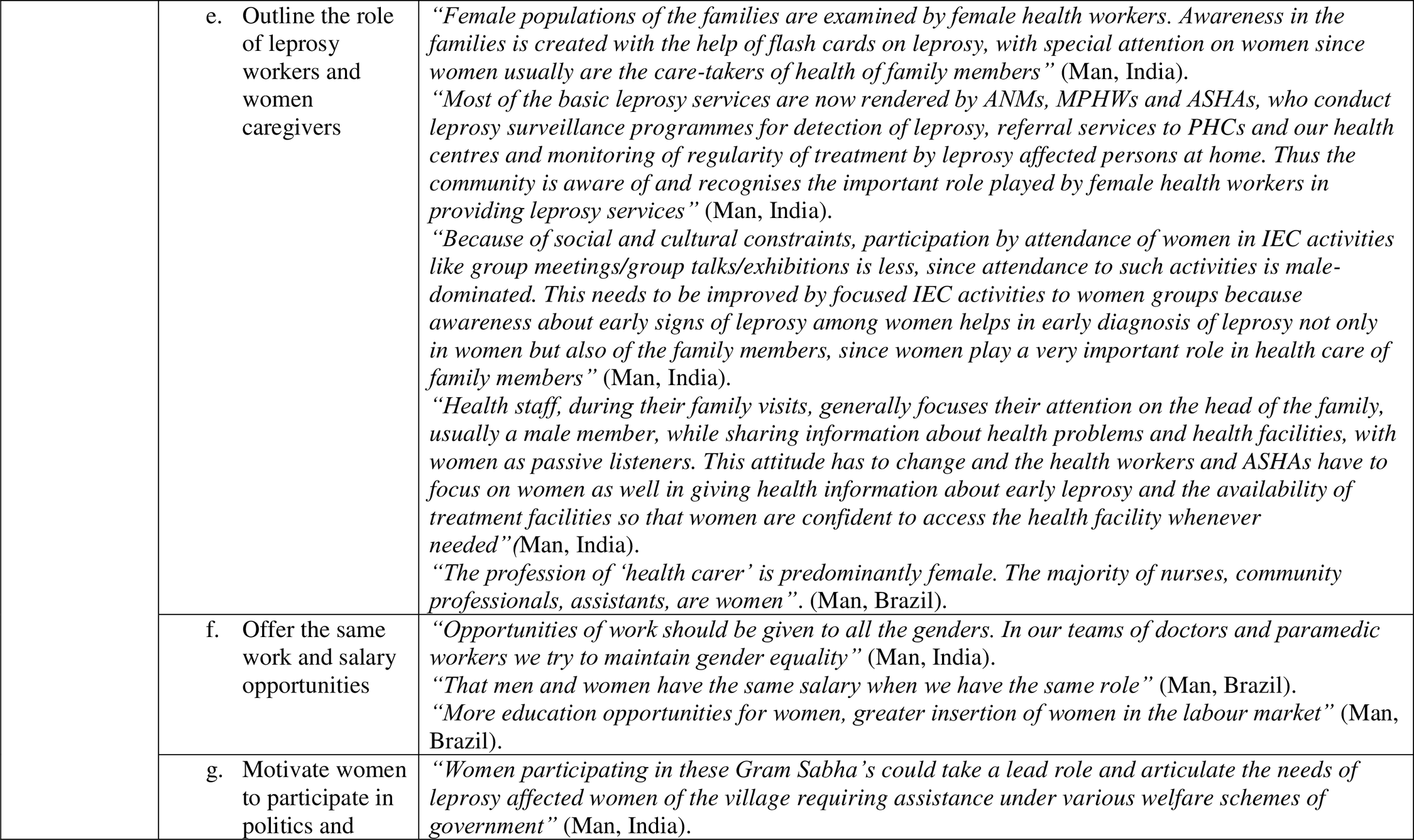

Supplement: S2 Table — (ZIP) [file pntd.0010335.s002.zip › PACE Corrected/S2_Table.tif]

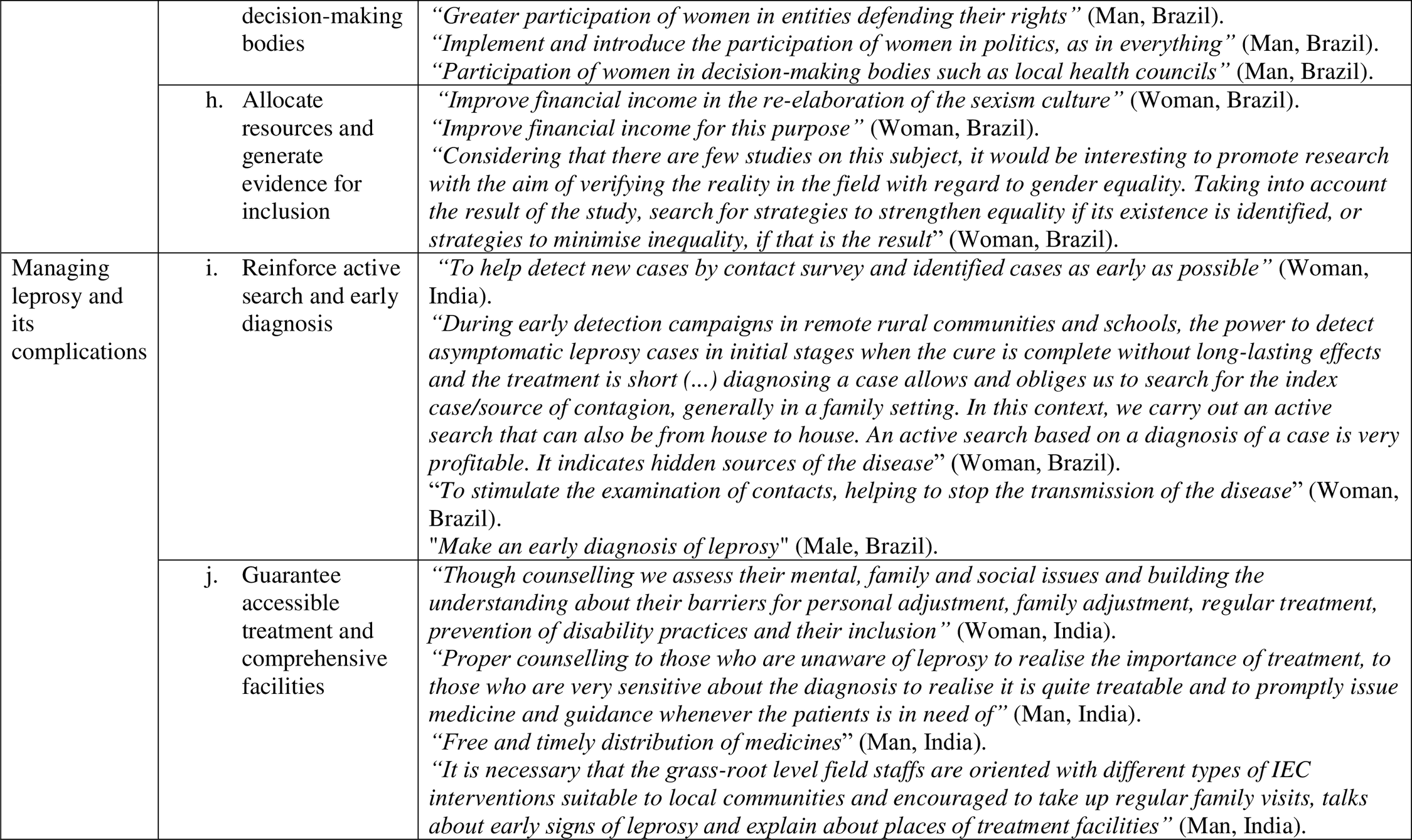

Supplement: S2 Table — (ZIP) [file pntd.0010335.s002.zip › PACE Corrected/S2_Table.tif]

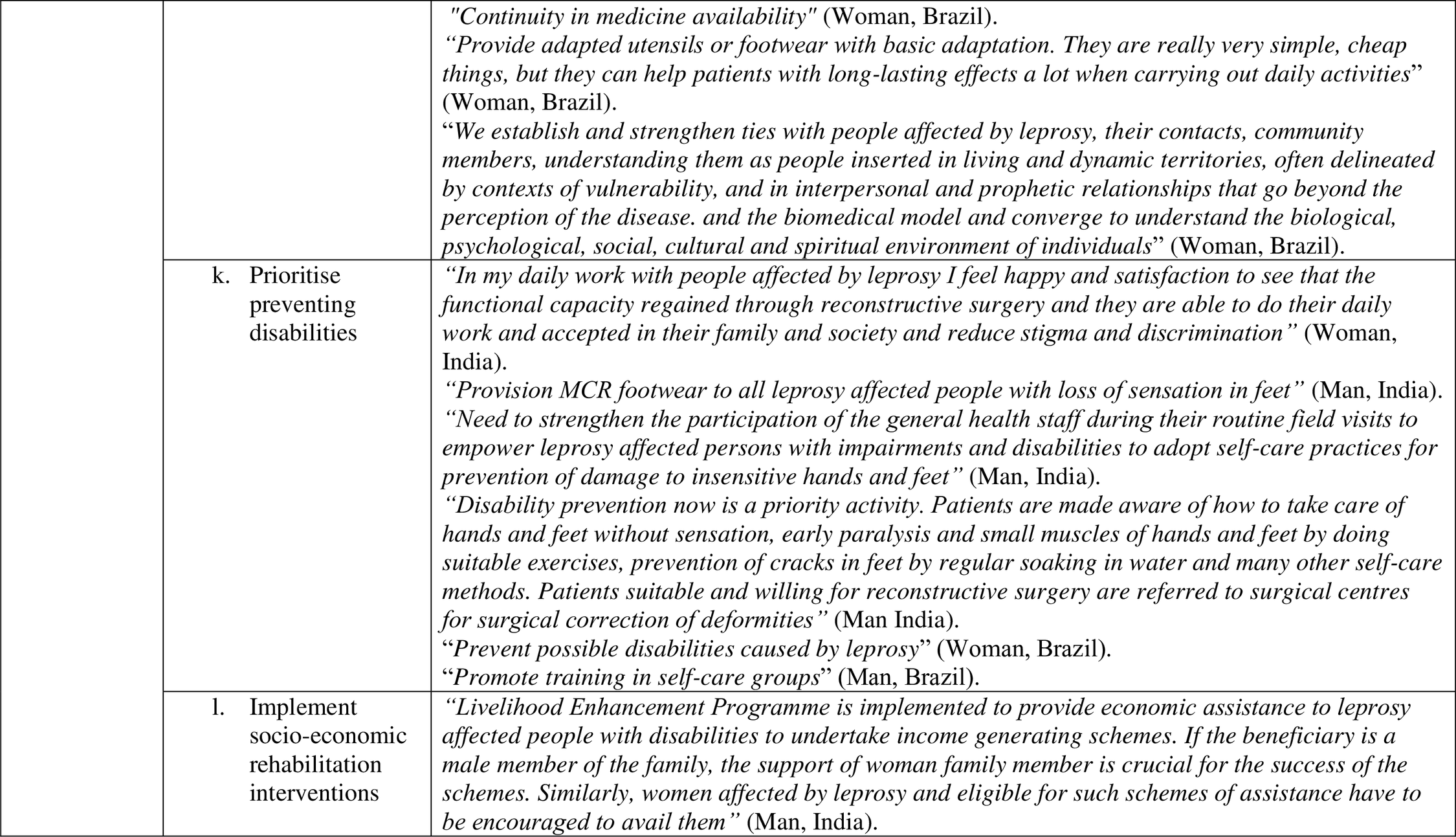

Supplement: S2 Table — (ZIP) [file pntd.0010335.s002.zip › PACE Corrected/S2_Table.tif]

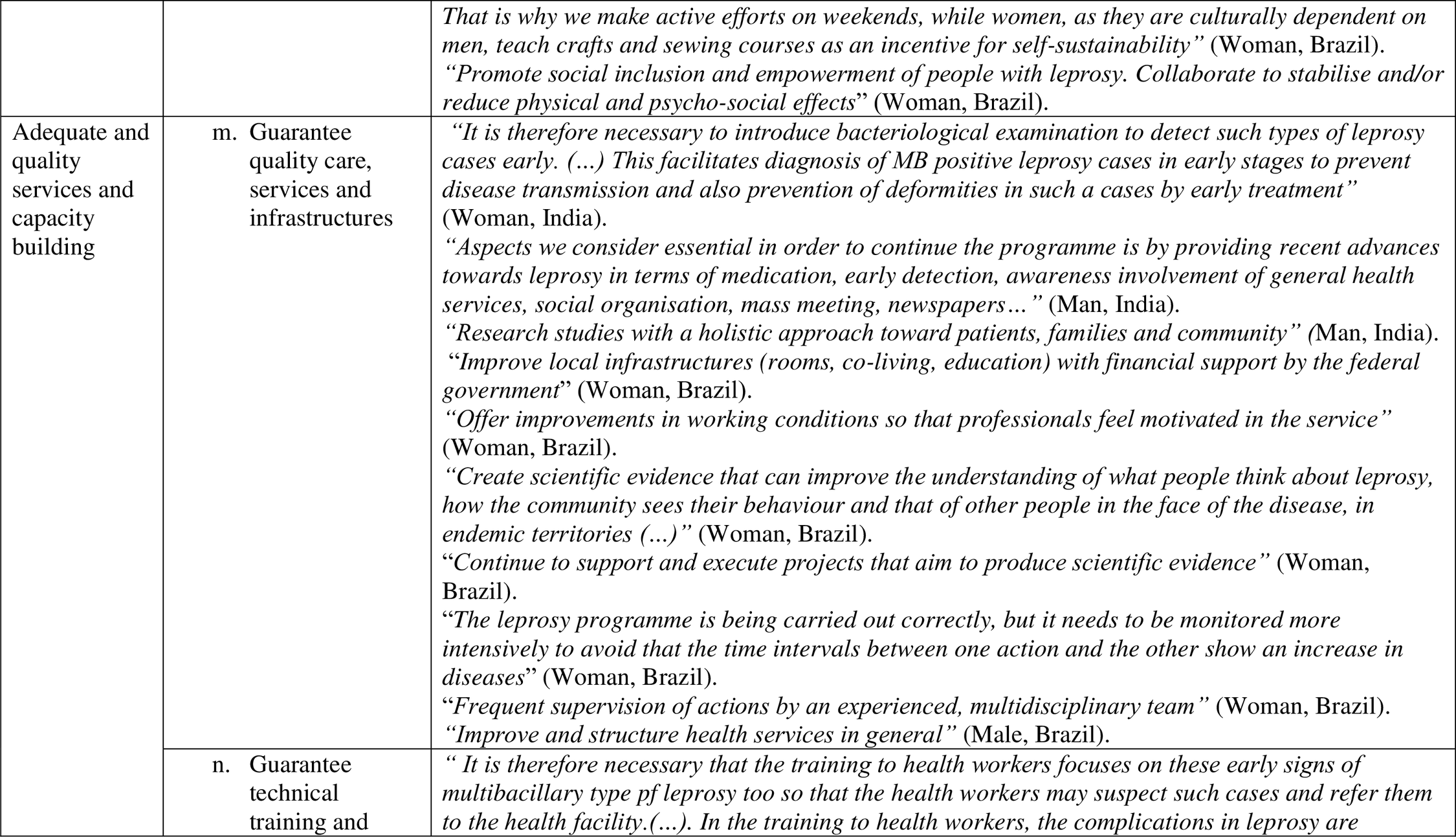

Supplement: S2 Table — (ZIP) [file pntd.0010335.s002.zip › PACE Corrected/S2_Table.tif]

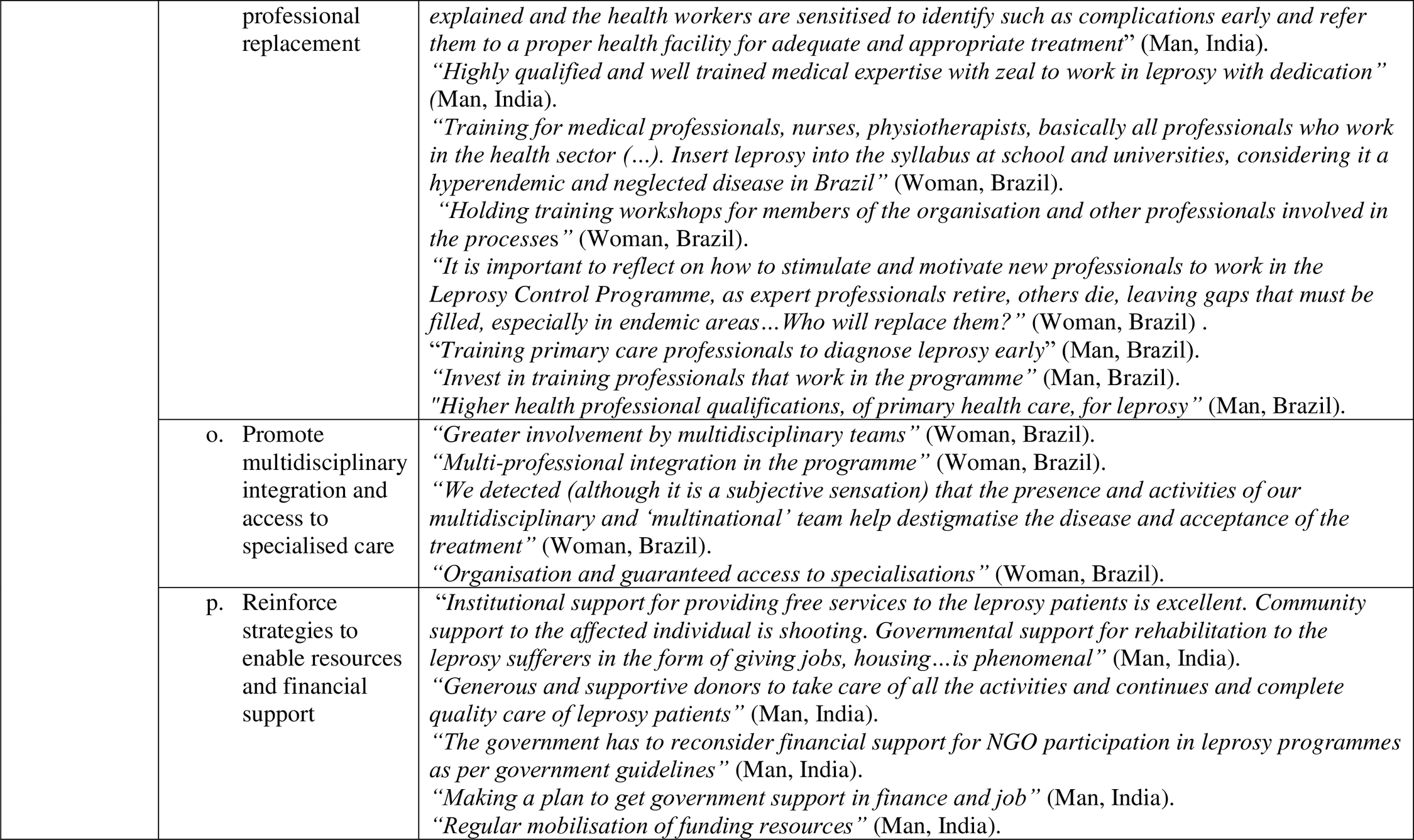

Supplement: S2 Table — (ZIP) [file pntd.0010335.s002.zip › PACE Corrected/S2_Table.tif]

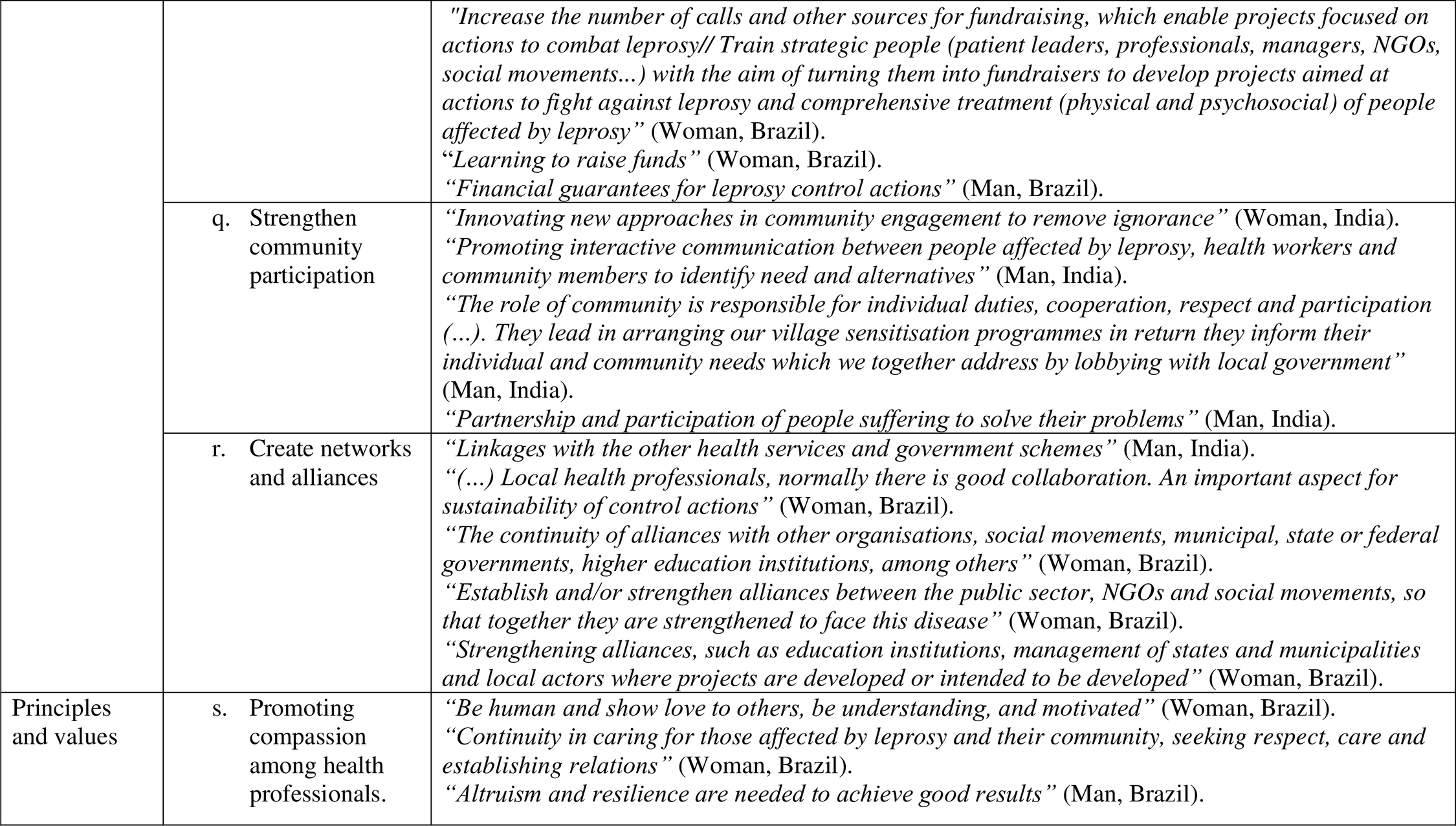

Supplement: S2 Table — (ZIP) [file pntd.0010335.s002.zip › PACE Corrected/S2_Table.tif]
